# *Notes from the Field:* Health Monitoring, Testing, and Case Identification Among Persons Exposed to Influenza A(H5N1) — Michigan, 2024

**DOI:** 10.15585/mmwr.mm7329a4

**Published:** 2024-07-25

**Authors:** Joseph Coyle, Natasha Bagdasarian, Seth Eckel, Jeremy Kuo, Mary Grace Stobierski, James Barber, Megan Weinberg, Fatema Mamou, Sarah Lyon-Callo, Michigan Local Health Departments, Bureau of Laboratories, Bureau of Infectious Disease Prevention Investigation Team, Michigan Local Health Departments, Jennifer Johnson, Dena Kent, Lisa Mikesell, Jennifer Morse, Becky Stoddard, Aimee Feehan, Chris May, Yolanda Rivera, Chad Shaw, Tamara Drake, Deral Glashower, Adeline Hambley, Gwen Unzicker, Rikki Fedewa, Susan Leeson, Clare Jansen, Kali Nichols, Maddie Vervaeke, Lisa Letts, Erin Radke, Elizabeth Baty, Darcie Cunningham, Kira Hecksel, Mary Huffman, Wai Yi Leung, Kassi Nelson, Sumeer Qurashi, Adenike Shoyinka, William Nettleton, Eric Pessell, Bureau of Laboratories, Katie Margulieux, Diana Riner, Marty Soehnlen, Jalen Stricklen, Jason Wholehan, Bureau of Infectious Disease Prevention Investigation Team, Smeralda Bushi, Derick Chia, Ebonē Colbert, Jim Collins, Justin Henderson, Tiffany Henderson, Shannon Johnson, Sue Kim, Mat Myers, Sarah Pruett, Briana Putrus, Bethany Reimink

**Affiliations:** 1 Michigan Department of Health and Human Services.; Mid-Michigan District Health Department; Mid-Michigan District Health Department; Mid-Michigan District Health Department; Mid-Michigan District Health Department; Mid-Michigan District Health Department; Ionia Health Department; Ionia Health Department; Ionia Health Department; Ionia Health Department; Ottawa County Health Department; Ottawa County Health Department; Ottawa County Health Department; Ottawa County Health Department; Central Michigan District Health Department; Central Michigan District Health Department; District Health Department #10; Barry-Eaton Health Department; Barry-Eaton Health Department; Allegan Health Department; Allegan Health Department; Ingham County Health Department; Ingham County Health Department; Ingham County Health Department; Ingham County Health Department; Ingham County Health Department; Ingham County Health Department; Ingham County Health Department; Ingham County Health Department; Calhoun County Health Department; Calhoun County Health Department; Michigan Department of Health and Human Services; Michigan Department of Health and Human Services; Michigan Department of Health and Human Services; Michigan Department of Health and Human Services; Michigan Department of Health and Human Services; Michigan Department of Health and Human Services; Michigan Department of Health and Human Services; Michigan Department of Health and Human Services; Michigan Department of Health and Human Services; Michigan Department of Health and Human Services; Michigan Department of Health and Human Services; Michigan Department of Health and Human Services; Michigan Department of Health and Human Services; Michigan Department of Health and Human Services; Bureau of Infectious Disease Prevention Investigation Team; Michigan Department of Health and Human Services; Michigan Department of Health and Human Services

**Summary**

**What is already known about this topic?**

Highly pathogenic avian influenza (HPAI) A(H5N1) virus has been detected in wild birds and mammals, poultry, and commercial dairy facilities in the United States. A human case in a Texas dairy worker was reported in April 2024.

**What is added by this report?**

As of May 23, 2024, Michigan had the largest number of affected dairy and poultry facilities linked to the HPAI A(H5N1) outbreak. Active symptom monitoring and testing of exposed workers led to detection of the second and third known dairy-associated HPAI A(H5N1) cases in 2024.

**What are the implications for public health practice?**

The current risk to the public from HPAI A(H5N1) viruses is low; however, continued symptom monitoring and testing are critical to characterizing genetic or epidemiological changes that might alter the risk assessment.

On March 25, 2024, a Texas dairy farm detected highly pathogenic avian influenza (HPAI) A(H5N1) virus in cows. The outbreak widely spread after interstate cow movement. During March 25–June 17, animals at a total of 102 dairy farms in 12 states, 24 commercial poultry flocks in five states, and multiple backyard flocks tested positive for HPAI A(H5N1) (*1*,*2*). This report describes response activities in Michigan, which led to detection of the second and third human cases related to the 2024 HPAI A(H5N1) outbreak. The activity was reviewed by the Michigan Department of Health and Human Services, deemed not research, and was conducted consistent with applicable federal law, state, and departmental policy.*

## Investigation and Outcomes

Infected cows from Texas resulted in introduction of HPAI A(H5N1) virus in a Michigan dairy, detected on March 29. As of May 29, a total of 23 Michigan dairies in 10 counties are known to be affected (*1*). Michigan’s first affected commercial poultry facility was confirmed on April 2; currently, seven affected poultry facilities in four counties have been identified (*2*). HPAI A(H5N1) virus has also been detected in a backyard flock, pigeons, foxes, cats, opossums, and a racoon in Michigan. Whole genome sequencing results suggest that, since March 2024, all sequenced isolates have ancestral Texas origins (*3*).

### Monitoring of Dairy Workers

Among the 23 affected dairies, 306 persons exposed to affected cows were identified. Lists of exposed persons were obtained by public health officials from 20 (87%) affected dairies. Workers at 12 (60%) of those dairies were enrolled in text-based daily symptom monitoring,^†^ and workers at eight (40%) farms were monitored through a farm point of contact. Because it could be unclear when workers’ exposures to cows ended, some workers were monitored for >50 days.

Twenty (6.5%) exposed workers reported symptoms and were tested for influenza A(H5) virus infection. Among persons who received real-time reverse transcription–polymerase chain reaction testing,^§^ one received a positive test result from a conjunctival swab, similar to the case of HPAI A(H5N1) reported from a dairy worker in Texas (*4*). Before the onset of mild unilateral conjunctivitis, the patient reported direct ocular exposure to raw, unpasteurized milk from an affected cow. A second worker from a different dairy farm experienced respiratory symptoms after close contact with sick cows and received a positive A(H5) virus test result from a nasopharyngeal swab. In both instances, public health officials rapidly collected patient specimens, which tested positive for HPAI A(H5N1). Neither worker was severely ill, neither required hospitalization, and no household or work contacts reported being ill. Both workers wore some personal protective equipment (PPE), but neither wore a mask or respirator.

### Monitoring of Poultry Workers

Among seven affected commercial poultry facilities, 857 persons exposed to affected birds were identified. Lists of exposed persons were obtained from all facilities. Workers from four facilities were directly enrolled in text-based daily symptom monitoring, and workers from three facilities were monitored through a farm point of contact who reported results to public health officials. Eighteen (2.1%) symptomatic persons were identified and tested; all test results were negative for influenza A(H5).

### Monitoring of Other Exposed Persons

Federal and state employees who responded to affected farms were also observed for symptoms, as were persons with exposure to HPAI A(H5N1) virus–infected animals (domestic or wild) or humans. Overall, 125 such persons were monitored, and 15 (12%) reported symptoms, 14 of whom received negative influenza A(H5) test results.

## Preliminary Conclusions and Actions

Among 1,288 Michigan residents who were monitored for signs and symptoms after potential HPAI A(H5N1) virus exposure, 53 (4.1%) reported signs and symptoms, 52 of whom received testing for influenza A(H5). Two dairy workers received positive test results (3.8% of all persons tested, <1% of all monitored dairy workers). 

Although the risk for HPAI A(H5N1) virus to the public remains low, novel influenza A viruses such as A(H5N1) have pandemic potential. Therefore, it is critical to notify persons with exposure to infected animals, provide education and access to PPE,^¶^ monitor signs and symptoms, test specimens collected from any exposed person with signs and symptoms, and make antivirals available to symptomatic persons as soon as possible.**

Although the percentage of workers who regularly used PPE is not known, the human cases associated with dairy farms in Texas and Michigan demonstrate the potential value of PPE, including eye and respiratory protection, especially on affected farms (*4*,*5*). The cases identified to date have resulted in mild illness, which might not have been detected without the collaboration of state officials and the engagement of farms and workers. Streamlined, nonintrusive approaches to monitoring, such as the text-message monitoring used in Michigan, might encourage participation and subsequent testing. A One Health^††^ approach including collaboration with agriculture departments, farms, and workers is crucial to successful public health response.

## Footnotes

* 45 CFR part. 46; 5 U.S.C. 301; 42 U.S.C. 289(a); 42 U.S.C. 300v-1(b).

†
https://people.health/

§ https://www.cdc.gov/bird-flu/php/severe-potential/?CDC_AAref_Val=https://www.cdc.gov/flu/avianflu/severe-potential.htm

¶ https://www.cdc.gov/bird-flu/prevention/hpai-interim-recommendations.html

** Antiviral treatment is recommended with oseltamivir as soon as possible for outpatients and hospitalized patients who have suspected, probable, or confirmed cases of human infection with novel influenza A viruses associated with severe human disease. https://www.cdc.gov/bird-flu/hcp/novel-av-treatment-guidance/?CDC_AAref_Val=https://www.cdc.gov/flu/avianflu/novel-av-treatment-guidance.htm

†† One Health is an approach that recognizes the interconnectedness of human, animal, and environmental health. https://www.cdc.gov/one-health/about/index.html
